# Histomorphometric Evaluation of Socket Preservation Using Autogenous Tooth Biomaterial and BM-MSC in Dogs

**DOI:** 10.1155/2021/6676149

**Published:** 2021-05-12

**Authors:** Jin-Hyun Kim, Puneet Wadhwa, HongXin Cai, Dong-Hyung Kim, Bing Cheng Zhao, Ho-Kyung Lim, Hyon-Seok Jang, Eui-Seok Lee

**Affiliations:** ^1^Department of Oral and Maxillofacial Surgery, Graduate School of Clinical Dentistry, Korea University, Seoul 08308, Republic of Korea; ^2^The CONVERSATIONALIST Club, School of Stomatology, Shandong First Medical University & Shandong Academy of Medical Sciences, Tai'an, Shandong 271016, China

## Abstract

This study is aimed at assessing the dimensional alterations occurring in the alveolar bone after premolar extraction in dogs with histomorphometric and histological analysis. After atraumatic premolar extraction, tooth-derived bone graft material was grafted in the extraction socket of the premolar region in the lower jaws of six dogs in two experimental groups. In the second experimental group, BM-MSCs were added together with the graft. The control was left untreated on the opposite side. After twelve weeks, all six animals were sacrificed. Differences in alveolar bone height crests lingually and buccally, and alveolar bone width at 1, 3, and 5 mm infracrestally, were examined. Histologic study revealed osteoconductive properties of tooth biomaterial. A statistically significant difference was detected between the test and control groups. In the test groups, a reduced loss of vertical and horizontal alveolar bone dimensions compared with the control group was observed. Tooth bone graft material may be considered useful for alveolar ridge preservation after tooth extraction, as it could limit the natural bone resorption process.

## 1. Introduction

Regenerative treatment research in dentistry has made major advancements in the field of alveolar ridge augmentation. Esthetic rehabilitation with dental implants can be performed provided that the sufficient alveolar bone structure with appropriate vertical height and horizontal width is available. Preservation of the alveolar socket is significant for alveolar ridge conservation following the tooth extraction [1]. Physiologic atrophy of the alveolar bone due to healing after extraction complicates implant installation; it also reduces predictability in the absence of bone augmentation surgery. Autologous bone graft is the gold standard for augmenting the alveolar ridge prior to oral implant placement [2]. Alveolar bone resorption increases the duration of treatment and stress for the patient and reduces the esthetics.

Recently, with the development of biological materials and imaging techniques, an autologous bone substitute is a prime focus of many researchers. Recently, many studies are using autogenous bone grafts in combination with platelet-rich fibrin [3] or recombinant human bone morphogenetic protein-2 [4, 5], etc., to evaluate enhanced bone regeneration effect. An autogenous tooth biomaterial can be easily prepared from an extracted tooth which was nonrestorable or a third molar. It is biocompatible and avoids recipient site morbidity. It can be useful as graft material because of its osteoconductive properties [3].

The alveolar process dimensional changes taking place after extraction have been widely studied [6, 7]. Following the extraction, the resorption of buccal and lingual alveolar bone can be examined radiographically within five weeks [8]. Alveolar bone volume preservation after extraction eases dental implant placement resulting in enhanced esthetic and functional results [2]. The decrease in the vertical and horizontal bone volume is the main structural change after extraction. The buccal bone plates are susceptible to more resorption in comparison with the palatal/lingual wall. The position of the buccal bone wall is taken into consideration for three-dimensional implant positioning. The most coronal portion of the buccal bone is a thin bony layer also known as bundle bone which disappears following extraction causing more resorption in the buccal bone as compared to the lingual wall [9]. Distinctive alveolar bone resorption is observed in the initial twelve weeks after extraction [10]. Physiological alveolar bone resorption is dependent on factors like the extracted tooth position, size of the defect, and adjoining bone composition. The magnitude and pattern of alveolar bone loss can show great individual variation [11]. Negative parameters affecting wound healing at the extraction sites comprise severe periodontal disease, traumatic extraction, and wound infection [12]. A periodontally compromised tooth depicts a significant amount of bone loss radiographically when compared with a fractured tooth [13]. Socket preservation using bone substitute biomaterial may have a better result regarding wound healing and prevent the resorption of alveolar bone. The material crystallization type can have a direct influence on its biodegradation. A good crystallization is displayed by sintered bioceramics prepared at high temperatures and are primarily degraded by an interstitial liquid-dependent process [14]. Earlier clinical studies concluded that nanocrystalline hydroxyapatite paste and bovine-derived xenograft when applied with a collagen membrane were followed by predictable peri-implantitis defect resolution [15].

Depending on the grafted biomaterial properties, autogenous tooth biomaterial affected bone regeneration activity. Tooth biomaterial underwent gradual resorption and was replaced by new bone of excellent quality through osteoinduction and osteoconduction [16]. A preliminary clinical study has shown that tooth biomaterial may serve as a bone graft substitute material, owing to its enhanced osseointegrating properties [16]. Clinical results suggest osseous integration and gradual bone graft resorption. Distinctive physicochemical properties of tooth biomaterial intend it to be used either with or without applying a barrier membrane. The data of some studies suggests that tooth biomaterial's physicochemical properties can enhance wound healing after extraction [3, 16]. It can be hypothesized that immediately after a tooth biomaterial paste is applied, a three-dimensional protein cover on the surface is built. It can lead to reduction in the inflammatory response thereby supporting the rapid osseous organization in the defect. Consequently, the natural alveolar bone resorption can be avoided up to some extent. While multinucleated giant cells slowly resorb other minihydroxyapatite particles [17–20], the resorptive cells are contemplated to directly incorporate tooth biomaterial.

Mesenchymal stem cells derived from bone marrow exhibit self-renewal properties and can differentiate into different types of cells [21, 22]. Bone marrow mesenchymal stem cells (BM-MSCs) help in bone formation by forming osteoblasts. The osteoprogenitor cells differentiate into preosteoblasts, which finally develops into mature osteoblast. These osteoblasts become osteocytes by encasing in the osteoid [23].

The present study is aimed at evaluating histologically and histomorphometrically the changes in ridge dimensions following tooth biomaterial application in the dog's extraction sockets. We analyse the alveolar crest height difference which is the position of the most coronal part of the buccal wall in reference to the most coronal part of the lingual wall. We used mongrel dogs in this study as they are less expensive and adequate for a short duration of experiments.

## 2. Materials and Methods

This study protocol was approved by the Institutional Animal Care and Use Committee of Korea University (KUIACUC-2010-168). Six adult mongrel dogs of either sex weighing approximately 25 kg were included in this study. All animals had fully erupted, healthy permanent dentition. In each animal, ridge preservation using tooth biomaterial and bone marrow mesenchymal stem cells (BM-MSCs) was performed after tooth extraction. BM-MSCs were isolated from the femur of each dog.

The study design was based on the Block and Kent study protocol presented in 1986 [24]. The premolars in the mandible were extracted as carefully as possible. The extraction sockets on one side were filled using tooth biomaterial, and the control side was kept unfilled, according to the study design. After 12 weeks, all animals were sacrificed, and specimens were collected for histologic and histomorphometric evaluation.

### 2.1. Isolation of BM-MSCs

Bone marrow (10 ml) was obtained from the iliac crest of each dog and washed with phosphate-buffered saline (PBS; SIGMA, St. Louis, MO, USA). The mononuclear cells in collagenase were then collected. After the cells were washed with PBS, they were resuspended in *α*-minimum essential medium (*α*-MEM; GIBCO, Grand Island, NY, USA) supplemented with 10% fetal bovine serum (FBS; Invitrogen, USA), 1% antibiotic-antimycotic solution (Gibco), and 4 ml L-glutamine (Invitrogen, USA), seeded into T75 flasks. Cultures were preserved at 37°C in a humidified atmosphere with 5% CO_2_. Approximately one to four weeks later, colonies containing fibroblast-like cells began to appear; these colonies were continuously subcultured for future experiments. After a confluent monolayer was formed, cells were passaged with the assistance of 0.05% trypsin/0.02% EDTA (Life Technologies Ltd., UK). A scanning electron micrograph of the ATP and BM-MSCs seeded onto tooth biomaterial was taken at 3, 7, and 21 days.

### 2.2. Surgical Procedure

Intramuscular sedation was done using 2.0 mg/kg xylazine (Rompun, Dai Han Parm, Seoul, Korea), and then, anesthesia was started with 10.0 mg/kg ketamine (ketamine hydrochloride, Dai Han Parm, Seoul, Korea). For postoperative pain relief, intramuscular injection of 0.4 mg/kg piritramide (Dipidolor, Seoul, Korea) and 4.5 mg/kg morniflumate (Morniflu, Seoul, Korea) was administered. For postoperative treatment to reduce the likelihood of infection, 10.0 mg/kg body weight cefminox sodium hydrate (Meicelin, Seoul, Korea) was given prophylactically, for three days.

All 8 premolar teeth (4 on each side; tooth position according to modified Triadan system: 305, 306, 307, 308, 405, 406, 407, and 408) of the mandible were extracted with care following vertical tooth separation. A total of *n* = 48 premolars were extracted. After extraction of 4 premolars of one random side, the teeth were processed by the Korea Tooth Bank as explained in an earlier study [3]. While the extraction sockets on one side were filled with tooth biomaterial alone in group 1 (*n* = 12), tooth biomaterial with BM-MSCs was used in group 2 (*n* = 12), and the extraction sockets on the other side remained unfilled, control group (*n* = 24). After, the vertical flap elevation wounds were closed with 3-0 absorbable sutures (Vicryl, Ethicon, UK). The animals were sacrificed following a healing period of 10 weeks. The mandible was dissected to procure the block consisting of the specimens from the experimental or control site. These specimens were fixed using 4% neutral buffered formalin solution for seven days. The dehydration of the samples was done with ascending grades of alcohol, then infiltrated and embedded in methacrylate resin for decalcified sectioning. All specimens were cut from the buccal to lingual direction with the extraction socket axis using a diamond band saw, creating three sections of approximately 200 *μ*m thickness. Each section was stained with hematoxylin and eosin after superficial etching and decalcification with 20% hydrogen peroxide and 10% formic acid.

### 2.3. Histological and Histomorphometric Analysis

**Figure 1:**
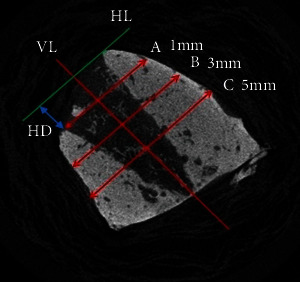
Histomorphometric evaluation of vertical parameters: height differences (HD) between buccal and lingual crestal bone. Histomorphometric evaluation of horizontal parameters: alveolar bone width at level 1 mm (A), 3mm (B), and 5 mm (C) infracrestally from HL (highest level), measured perpendicular to the long axis or vertical line (VL) of the extraction socket.

Histomorphometric and histological examination was executed by a competent examiner, who was blinded to the experimental settings. Digital images were evaluated with the help of a software program (Imagescope Version 9.1.19.1571; Aperio Technologies, CA, USA). The extraction socket evaluation was done by measuring the total bone height of lingual and buccal calculated from the top of the buccal and lingual alveolar wall to the most apical point of the jaw; buccolingual alveolar bone wall width was calculated at 3 levels A, B, and C perpendicular to the long axis or vertical line (VL) of the extraction socket marked at 1 mm (A), 3 mm (B), and 5 mm (C) infracrestally from HL (highest level) which is the highest point of alveolar bone and perpendicular to VL (Figure 1).

### 2.4. Statistical Analysis

For statistical analyses, the buccal and lingual alveolar crest height differences were calculated. The SPSS 14.0, SPSS Inc., Chicago, IL, USA, software was used for statistical analysis. We used the Wilcoxon tests for inductive statistics between the test and control sites of each animal. Differences were considered statistically significant at a level of *p* < 0.05.

## 3. Results

**Figure 2:**
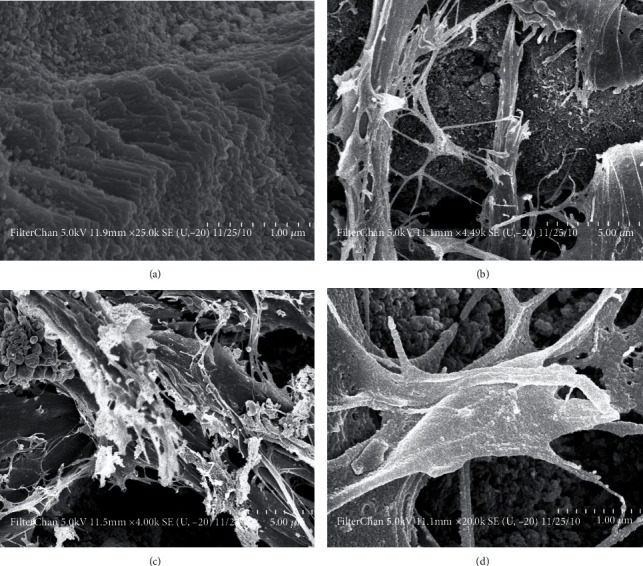
(a) Scanning electron micrograph of the tooth biomaterial; (b) BM-MSCs seeded onto tooth biomaterials at 3 days; (c) BM-MSCs seeded onto tooth biomaterial at 7 days; (d) BM-MSCs seeded onto tooth biomaterial at 21 days.

All extraction sockets healed without any adverse effects. There were no observed signs of infection or wound dehiscence during the entire study duration. A scanning electron micrograph (SEM) of the ATP and BM-MSCs seeded onto tooth biomaterial was taken at 3, 7, and 21 days. The adhesion ability of BM-MSC on the tooth biomaterial as well as the biocompatibility was observed under SEM. It was confirmed that the cells were well spread and adherent to the graft material (Figure 2).

### 3.1. Histological Analysis

**Figure 3:**
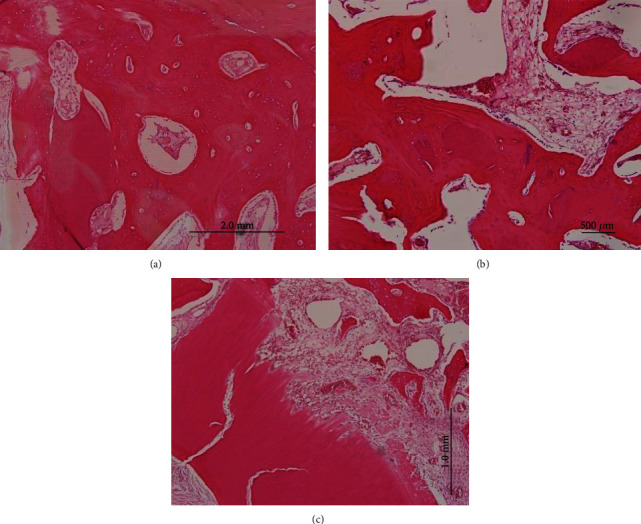
(a) Histological specimen of the tooth biomaterial site at 12 weeks: the new bone tissue covers the defect with compact bone and closing the alveolar crest (original magnification 12.5x); (b) defect filled by high calcified bone tissue: calcified bone formed an interconnected system with the alveolar wall (original magnification 40x); (c) Howship's lacunae are observed on the periphery of tooth biomaterial, indicating continuing tooth biomaterial resorption (original magnification 200x).

With tooth biomaterial of different specimens, the resorption of the graft and histological appearance varied. The experimental groups 1 and 2 were almost filled with tooth biomaterial or tooth biomaterial with BM-MSCs; few specimens demonstrated medium or high-grade tooth biomaterial resorption (Figure 3(c)). In group 1, in the crestal region, horizontal bone bridges were observed creating a gap between tooth biomaterial areas. On the alveolar wall near the surface of the extraction site, a layer of compact bone without any blood vessel penetration was noticed (Figure 3).

**Figure 4:**
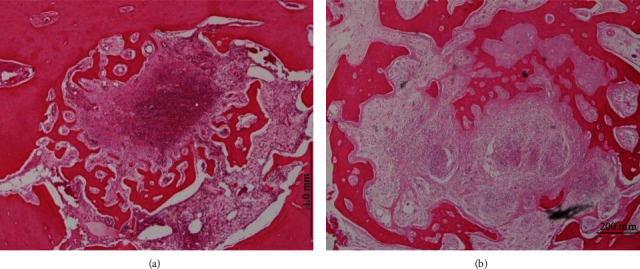
(a) Histologic sections of the control site at 12 weeks (original magnification 12.5x). The soft tissue defect remained; compact bone absence ceases the closure of the alveolar crest. The cancellous areas are still in contact with the soft tissue; (b) higher magnification of (a): minor calcified bony tissue fills the defect, and cancellous bone beneath the alveolar crest is noted (original magnification 100x).

When tooth biomaterial was observed under higher magnification, it presented colored areas of different intensities. On the experimental side groups 1 and 2, the tooth biomaterial was encased by newly formed bone with indications of crystallization beneath the little bony layer, revealing osseointegrated fragments. On the control side, the extraction sockets were left empty. Intense staining of hematoxylin and eosin was observed indicating lowered calcification of the new bone formed. The spongiosa areas of the alveolar crest came in contact with the soft tissue, although the remodeling of the alveolar crest with compact bone was assumed to be making progress. The alveolar surface exhibited higher Howship's lacunae (Figure 4).

**Figure 5:**
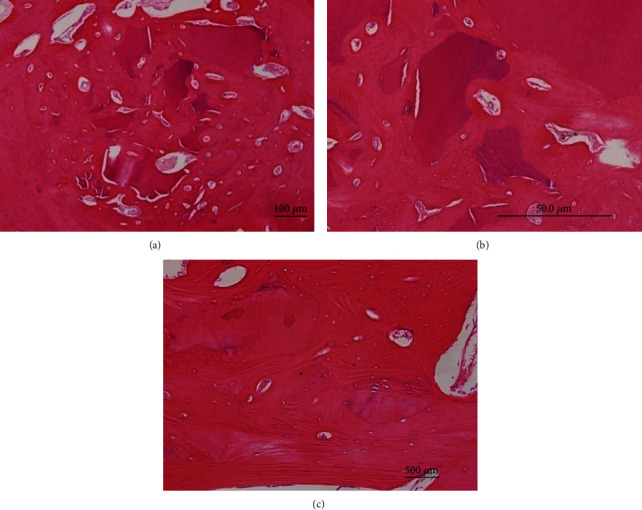
(a) Tooth biomaterial and BM-MSC specimen at 12 weeks: the bone defect was almost filled with tooth biomaterial remains and new bone; (b) higher magnification of the alveolar socket: augmented areas are composed of new bone covering new bone bridges. (c) The hard and soft tissue healing with a compact bony layer structure on the alveolar crest: the tooth biomaterial demonstrates osteoconductive effect with the presence of newly formed bone.

After 12 weeks in group 2, the specimens of tooth biomaterial showed that the defect areas were covered by layers of compact bone. Few remnants of tooth biomaterial were observed due to high resorption rates (Figure 5). Tooth biomaterial particles were still enclosed by bone tissue. In the control group, the newly formed bone and the old tissue could not be differentiated well. The defects were incompletely ossified, and a round compact bony layer along with normal gingival tissue wrapped the cancellous bone at the crestal region (Figure 4).

### 3.2. Histomorphometric Analysis

Means and standard deviations of bone height difference and the alveolar wall thickness measurement are presented in Tables 1 and 2. The bone height differences showed significantly higher value in the experimental groups 1 and 2 than the control group (*p* < 0.05, Wilcoxon test). The alveolar wall thickness parameter did not reveal any statistically significant difference between the experimental and control sites in each animal (*p* > 0.05, Wilcoxon test).

## 4. Discussion

After extraction of a tooth, alveolar ridge changes occur in the horizontal and vertical directions [25]. A few histological studies on the tooth biomaterial used here can be found in the literature [26]. In our study, the bone graft material did not cause any adverse inflammatory reactions, osteofibrosis, or osteonecrosis. The varying calcification rates may be responsible for the variation in coloration of the particles, lower the rate of calcification, and higher the intensity for mineralized tissue staining. The particles exhibiting a highly intense surface staining could be due to resorption instigated by surrounding tissue [27]. The new crystallization nucleus existence detected at the transitioning layer with the adjoining bone suggests that the decalcified area on the osseointegrated surfaces contributes to forming new bone. These histological observations emphasize that for new hard tissue formation, the tooth biomaterial should resorb completely. Tooth biomaterial remnants were detected in a human study for peri-implantitis defect bone grafting [16]. In the histological sections, most of the tooth biomaterial was osseointegrated with new bone after eight weeks. In our study, specific stained sections showed that multinucleated giant cells and macrophages actively resorbed some tooth biomaterial remnants as shown in an earlier study [16].

In the present study, extraction socket defects were filled using tooth biomaterial and BM-MSCs. The microradiographic and histologic evaluation suggested significantly higher rates of mineralization in the experimental groups as compared to the control group. The effect of the experimental design must also be considered. The variation in the shape of the target area may also be a factor for a slower resorption rate. In many other studies, the applied bone graft was confined within the acute voids amid bony walls while the shape of the defect after a dental extraction relates to the root configuration, resulting in a thicker crestal region. The blood component primary invasion depth into the hydroxyapatite suspension might be of importance in the consequent graft material osseous organization. Some studies suggested inhibition of blood vessel growth and bone formation with granular hydroxyapatite pore size less than 100 *μ*m [28, 29].

Shrinkage of the graft material during primary wound organization or the periodontal ligament cell impact must be taken into consideration. Some authors examined the periodontal ligament role in healing after extraction, suggesting that the periodontal ligament may not play an important role in the healing process [30]. There was no difference between socket preservation with tooth biomaterial and tooth biomaterial with BM-MSCs within the healing period of three months. A reduction in vertical degeneration of the alveolar ridge in comparison with the control was observed following the use of preformed root-like hydroxy tapers, particulate hydroxyapatite, and *β*-tricalciumphosphate root tapers in other animal studies [31, 32]. Although the *β*-tricalciumphosphate granules resorbed within 60 weeks, the extraction socket regeneration seemed to be slackened in comparison to the control group [12]. This deceleration detected is in accordance with the present findings, as the control exhibits complete hard tissue healing after a period of six months, but not the experimental group. The use of membrane either with [33] or without a filling material support [34, 35] could prevent dimensional changes following tooth extraction. In another clinical study, when polytetrafluoroethylene membranes were used immediately after extraction, after six months, significantly smaller alveolar ridge dimensional changes were observed as compared to untreated extraction sockets [34]. The use of polylactide/polyglycolide membranes led to comparable results, despite early membrane exposure causing a critical drop in the clinical outcome [35]. A significant amount of buccal resorption corroborated the results of another extraction study [25]. In an animal study, immediately after extraction, a screw-shaped titanium implant was inserted, but the buccal resorption could not be avoided [36].

## 5. Conclusion

In this study, no statistically significant difference was observed in alveolar height difference or alveolar wall thickness between tooth biomaterial graft groups with or without BM-MSC. Within the limits of the present study, it was concluded that tooth biomaterial, showing evidence of osseous integration, is a useful graft material for socket preservation procedures.

## Figures and Tables

**Table 1:** Means and standard deviations of bone height differences.

	Tooth biomaterial	Tooth biomaterial+BM-MSCs	Control
Height (mm)	1.7 ± 1.2	1.6 ± 1.0	3.5 ± 0.8

**Table 2:** Means and standard deviations of alveolar wall thickness at different levels.

	Tooth biomaterial (*n* = 12)	Tooth biomaterial+BM-MSCs (*n* = 12)	Control (*n* = 24)
Level A (mm)	3.3 ± 1.0	3.5 ± 0.9	2.4 ± 0.7
Level B (mm)	5.4 ± 0.4	5.6 ± 0.5	5.1 ± 0.3
Level C (mm)	6.5 ± 0.3	6.7 ± 0.6	6.4 ± 0.4

## Data Availability

The data used to support the findings of this study are available from the corresponding author upon request.
